# Camp Dysentery

**Published:** 1861-08

**Authors:** C. D. Griswold

**Affiliations:** Cleveland, Ohio


### CAMP DYSENTERY.

BY C. D. GRISWOLD, M.D., Cleveland, Ohio.

MALARIOUS ORIGIN—PATHOLOGY COMPARED WITH THAT OF MALARIOUS FEVERS—"ABORTIVE" TREATMENT OF DYSENTERY, &C.

Dysentery is the plague of the army. In hot climates it often proves more destructive to the life of the soldier than the enemy's projectiles. It is the foe the commander most dreads to see appear, for no skill in tactics, no advantage in position, or bravery of his forces, avails in the contest. With the appearance of dysentery in an epidemic form, the General is sometimes compelled to resign his command into the hands of the Surgeon, and wait events. It was this disease that decimated the French army so terribly in Egypt, the English armies in India, and our own in Mexico. Wherever large bodies of men are compelled to endure great fatigue during the heat of the day, sleep on the ground at night with only a blanket between them and the earth, and to cover and protect them from the dew and night air, there dysentery in its epidemic form is almost sure to appear sooner or later. The only other circumstances necessary to make it *certain*, perhaps, are a near approach to the tropics, or within them, and camping on low grounds.

*Cause of Dysentery.*—Epidemic dysentery, it is believed by many systematic writers, has its origin in malaria. While in charge of the hospital as surgeon of the Panama Railroad Co. at Rujio Saldado, during the construction of that road on the Isthmus, I saw much of this disease, and was led from its behavior to regard it as essentially a malarious disease. During the rainy season, corresponding with our summer, when the men on the works were subject to drenching showers, and would go into their quarters, and perhaps lie down without taking off or changing their clothes, to awake in the night chilled by the cool air, dysentery became the prevailing disease. On the other hand, when drought prevailed, we had fever alone to combat. The treatment sustained fully the malarious nature of the disease. Opium and its concomitant adjuncts had little control over the discharges, but if possible they were stayed by such means, a chill would set in followed by fever, to arrest which, quinine would be administered and the patient would recover as though no dysenteric symptoms had preceded it; but such a course would protract the case through several days; a matter of great importance, not only to the patient but the Company, inasmuch as the average expense of each man was five dollars a day. Where ninety out of one hundred were taken down with either dysentery or fever, within two weeks after arriving upon the Isthmus, it became a matter of the utmost importance to return the sick to their work in the shortest possible time. To this end we soon learned that quinine was as essential in cutting dysentery short as it was in accomplishing the same end in the treatment of the intermittent and remittent fevers—thus sustaining the opinion as to its malarious origin, instead of regarding the fever as a complication, as some would have it.

During a residence of five years at Fort Hamilton, R. I., I had frequent opportunities of observing and treating dysentery. This district is perhaps one of the most pernicious localities for miasmatic fevers in the vicinity of New York, and here I found dysentery as readily cut short by quinine as on the Isthmus. In 1859, epidemic dysentery prevailed to a considerable extent in Batavia and the surrounding towns in Genesee Co., N. Y., where I then resided. It proved very fatal under the usual treatment, the patients sinking ultimately into a typhoid state. Here, as elsewhere, I found that quinine would bring the hæmorrhagic discharges to an early termination, and establish convalescence. Here dysentery was associated with a severe form of remittent, usually called typhoid fever, and which without the administration of quinine in many cases proved fatal.

From these observations thus briefly sketched, of the behavior of dysentery treated with and without quinine, together with the fact that it is always associated with some form of miasmatic fever, I have become satisfied that the agency of malaria as its cause is unquestionable. Its sporadic existence is not more ftequent or difficult to explain than that of intermittent fever.

*Pathology of Dysentry.*—Those who remember the papers I contributed some years ago to this Journal, know that I regard intermittent, remittent and yellow fever, as all having one common or malarious origin. Spring malaria produces intermittent; mid-summer malaria, remittent of a simple type; later in the season, gastric, bilious and typhoid symptoms complicate it, upon which may be engrafted the virulent malaria of yellow fever from southern climates. Such was the case at Fort Hamilton and Staten Island in 1856. But little intermittent had prevailed that spring, but in July a severe form of remittent set in, with marked gastric and bilious symptoms, upon which the yellow fever poison became engrafted and proved very fatal, except in cases where quinine was promptly and largely given on the first remission. Now these diseases are produced by a poison developed from inanimate matter, and that only, and therefore not contagious in any sense of the term. The same law and conditions pertain to dysentery, and when we add it, to the list, we have the principal malarial diseases grouped together.

Let us glance at the phenomena exhibited in the different forms of disease we have classed together as arising from a malarious origin. In simple ague—the type of all grades of fever—we have during the chill an exsanguinous surface, with engorgement of the lungs principally, and to a great degree suspension of the development of animal heat. In remittent fever, we find the lungs not so generally involved, but the liver, spleen and stomach take on the congestion from the receding venous currents. The symptoms are gastric and bilious, and the general constitution is more seriously affected. In that form of fever regarded by many writers as idiopathically typhoid, we have still less chill, with greater depression of the vital powers, and subsequent ulceration of Peyer's glands, indicating a still more deep-seated congestion in the early stages of the disease. In dysentery, the primary congestion or engorgement takes place near the terminal extremity of the intestinal tract—in the rectum and colon, and as these organs are not so important in nutrition, the constitutional symptoms are not usually so depressing or dangerous. In yellow fever, the primary engorgement involves almost every interior organ essential to organic life, especially the liver, stomach and intestinal tract. In extreme cases, unless the virulent poison of the malaria is early neutralized by quinine, the venous engorgement is not relieved, the vitality of the blood is suspended, and it transudes into the mucous cavities and is ejected from the stomach as black vomit.

A little careful reflection will show that in each form of disease referred to, the symptoms and constitutional effects are in perfect accordance and harmony with the lesion, and that their distinguishing characteristics are from local causes, rather than from any radical difference in the primary source —that dysentery is just as truly a malarial fever as intermittent, its widely different symptoms being the result of the lession, or seat in which the malarial poison takes effect. Why malaria in the spring should take effect principally in the chest; in the summer in the liver and stomach; and in the autumn in the small intestines or lower bowel, is more than I can tell, but the length of time the surface which eliminates the poison has been exposed to the sun before it becomes sufficiently dry to generate the malaria, seems to have much to do with it. In the former papers referred to above, I endeavored to show that vegetable decomposition in no case generates malaria, a conclusion which extended observation I believe will fully confirm.

*Treatment of Dysentery.*—Life in the camp is specially calculated to produce dysentery, or the local determination of blood to the lower bowels, when the primary cause is taking effect on the system. The hips are more frequently exposed to the ground, and without covering, than any other part of the trunk, while the recumbent posture upon the ground is most favorable to the inhalation of malarial poison. The soldier subject to such habits requires a treatment specially adapted to his situation. He should take nothing calculated to increase his susceptibility to change of temperature, nor to impair his vital forces. To equalize the circulation, instead of abstracting by blood-letting from it, should always be the rule. No error in practice is more frequently advised in text-books than this of bleeding in dysentery. Fortunately, from necessity, it is generally, I believe, dispensed with in the camp. Bloodletting was never beneficial in diseases involving the mucous membranes, and its disuse is rapidly following a better knowledge of the pathology of dysentery.

Mercury is another remedy which has been highly esteemed in dysentery, from a confused idea that this disease was in some way a sequence of diseased condition, or deranged function of the liver. All careful observers will find the hepatic difficulty but an *effect* and not a *cause* in dysentery, as in all malarial fevers. The primary effect of malaria is upon the organic system of nerves, and hence the processes of nutrition are suspended. The liver being the largest secretory gland in the system, the effect in this organ is most apparent. Malaria serves to lock up all the secretions, and hence the true remedy is that which will neutralize this poison, when all the organs resume their functions and the system returns to health. The use of mercury in dysentery is not indicated by any condition of the disease, and in camp dysentery it cannot be too freely deprecated. Its effect upon the constitution when long continued is analogis to that of malaria—the destruction of red globules in the blood, It is irritating to the mucous surfaces when diseased, and serves no purpose that may not be attained by the most simple cathartic. Mercury and malaria cannot be endured in the same constitution without a two-fold injury to the vital powers. Bloodletting and mercury are good remedies when inflammation involves serous membranes, but when the mucous tissue is diseased, abstain from their use. The liver will take care of itself if the primary cause of the disease is properly attended to.

Alternate cathartics and opiates is a favorite course with the old physicians. I have seen a patient sink and die within three days from the effects of a cathartic, and this, too, after the physician had decided convalescence to be fully established. The practice which I have found pre-eminently successful consists mainly in the administration of powders composed of Ind. rhei, grs. ij.; opii and ipecac., aa gr. ss. In preparing these powders I triturate the ingredients thoroughly in a mortar, combining a little white sugar. When there is indication of acetous fermentation in the stomach and bowels, add a little sub. carb. soda. When there is obstinate constipation, increase the rhubarb; if the bowels are relaxed, lessen it. If fever runs high, increase the ipecac. until nausea is produced. The rhubarb will work its way through the bowels and carry the opium to the seat of the disease, preventing the formation of scybala or the necessity of other cathartic remedies. When there is no fever at the commencement, I give quinine to the adult in four-grain doses, alternating with the above powder, every third hour. When there is well-marked pyrexia, wait until a distinct remission before giving the quinine; it should be continued until 16 to 20 grains are taken, or cinchonism is produced. With this simple treatment, commenced early in the disease, the great majority of cases will be cut short within forty-eight hours, and the patient may be permitted to go about his business. On the Isthmus a patient was seldom detained from his work more than one day from an attack of dysentery; and in simple ague he was merely required to report himself at the hospital before each meal, to take his quinine and go to his work regularly, in the majority of cases. The proportion of deaths to the whole number of cases on the Isthmus was very small, the statements of the secular papers to the contrary notwithstanding. The whole number during the first and second year was about 35. I have seen the number stated more than the whole number of persons, both native and foreign, ever employed upon the road from its commencement to its completion.

A wide-spread prejudice obtains with the public against the use of quinine, and many physicians do not seem to be much better informed upon the subject. It is as harmless, in producing any permanent constitutional effects, as capsicum. I have administered over a hundred ounces from first to last, without ever discovering any injurious effects from it. During the yellow fever epidemie at Fort Hamilton, L. I., and during and subsequent to an attack of that disease, I took about two hundred grains of it, and have never been in better health in my life than since. It should be remembered, however, that I was a month almost constantly breathing the poisoned atmosphere of that district, having no time to attend to myself as I did others, taking the remedy at hap-hazard as I felt the indications coming on. Quinine alone has no permanent effect in preventing the recurrence of fever. It simply neutralizes the poison of malaria for the time being. More permanent results were obtained from the use of the bark in powder, as administered before sulphate of quinine came into use. A concentrated preparation of the calisaya bark should always be used after breaking up a fever with quinine, in private or hospital practice.

The use of spirits by those subjected to a malarious atmosphere is exceedingly prejudicial. There are few constitutions that can bear up under the combined effects of malaria and strong drinks; fever is hastened and rendered less amenable to treatment by such practice. On the Isthmus this was most thoroughly tested, not only among the laborers, but with the people generally. Of the four deaths that occurred in my department, two of them were from this cause.

Many measures may be adopted by the physician or army surgeon not indicated here, for the relief of the patient suffering from dysentery; my object being to establish general principles for the "abortive" treatment of the disease, and not to dwell upon the minutiæ. If I have succeeded in calling attention to, and pointing out the way by which the miasmatic origin of dysentery may be recognized, I am confident that it will lead to a more speedy and successful treatment of this disease than that generally advocated by text-books and the profession.—*Bost. Med. Jour.*

